# 

**Published:** 1853-06

**Authors:** 


					﻿THE
WESTERN JOURNAL
OF
MEDICINE AND SURGERY
EDITKD BY
LUNSFORD P. YANDELL, M. D.,
PROFRflOY er PRYflOLOOY AND FATMOLOtl&AL ANATOMY IN TDK VNlVKRSfl Y OF LOViSYI1>l «,
AND
THEODORE S. BELL, M. D.
CLXIL—JUNE, 1853.
THIRD SERIK S
Vol. XI...No. VI.
LOUISVILLE, KY:
PUBLISHED MONTHLY BY PRENTICE &, HENDERSON
PROPRIETORS AND PRINTERS.
1 8 5 3.
				

